# 

**Published:** 1904-10-29

**Authors:** 


### NOTICE TO CORRESPONDENTS.

All MSS., Letters, Books for Review, and other matters intended for the Editor should be addressed The Editor, "The Hospital," The Hospital Buildings, 28 & 29 Southampton Street, Strand, W.C.

The Editor cannot undertake to return rejected MS., even when accompanied by stamped directed envelope.

**Notice to Advertisers and Subscribers.**

All Advertisements, Orders for Copies of The Hospital, and Business Communications should be addressed to **THE MANAGER (Not to the Editor),** The Hospital, 28 & 29 Southampton Street, Strand, London, W.C.

The Cost of Subscription, post free, is as follows:—Per week, 3½d.; per quarter, 3s. 3d.; per half-year, 6s. 6d.; per annum, 13s. Foreign and Colonial:—Per annum, 19s. 6d., payable, in all cases, in advance.

**NOTICE.—Vol. XXXVI. of "The Hospital" (April to September 1904), in handsome cloth binding, is now on sale at the office of this Journal, price 10s. 6d. Cases for binding the Numbers contained in this Volume 2s. Index to Vol. XXXVI., 3½d., post free.**

**The Monthly part for November will be ready on November 1st. Price 1s., by post, 1s. 3d.**

### BOOKS RECEIVED.

John Bale, Son, and Danielson, Ltd.
"Notes on Assouan, Egypt." By G. Dundas Edwards.

Rebman, Ltd.
"Diseases of Women." By Bland-Sutton and Giles.

E. Arnold.
"Lectures on Diseases of Children." By Robert Hutchison.

S. W. Partridge and Co.
"The Zenana, or Woman's Work in India." Vol. XI. 1904.

W. B. Clive (University Tutorial Press).
"Matriculation Directory," No. 38, September 1904.

### Medical Appointments.

**W. Alexander**, M.D., Medical Officer of Health, Branksome Urban District. **William Cairns**, M.B., M.S.Glasg., Health Officer for the Port of Whitianga, New Zealand. **J. Chestnutt**, B.A.Irel., L.R.C.P. and S.Edin., Medical Officer to the Post Office officials in Howden and district. **W. T. Clarke**, M.D., Toronto, Clinical Assistant, Chelsea Hospital for Women. **A. Coleridge**, M.R.C.S., L.R.C.P., Clinical Assistant, Chelsea Hospital for Women. **Arthur M. Cudmore**, F.R.C.S., L.R.C.P.Lond., Honorary Surgeon to the Adelaide Hospital. **T. A. Dillon**, L.R.C.P. and S.Irel., Resident Assistant Medical Officer, Croydon Union Infirmary. **A. W. Denny**, M.R.C.S., L.R.C.P., District Medical Officer of the Newmarket Union. **C. A. H. Gee**, M.R.C.S., L.R.C.P., District Medical Officer, Shepton Mallet Union. **A. W. Hill**, M.D., Ophthalmic Surgeon to the Adelaide Hospital. **R. M. Littler**, F.R.C.S., etc., Honorary Assistant Medical Officer to the Southport Infirmary. **Frank Magarey**, M.B., Ch.M.Syd., Assistant Surgeon to the Adelaide Hospital. **R. S. Rogers**, M.A., M.D., Honorary Physician to the Adelaide Hospital. **J. H. Stormont**, M.R.C.S., L.R.C.P., District Medical Officer, Solihull Union. **A. M. Ware**, M.D., Clinical Assistant Chelsea Hospital for Women.

**"The Hospital" Institutional Library.**

"Hospitals and Asylums of the World." 4 Vols., with a Portfolio of Plans, complete £12 12s.

"Burdett's Hospitals and Charities, 1904." 5s. net.

"Burdett's Official Nursing Directory." 8s. net.

"Cottage Hospitals, General, Fever, and Convalescent." 10s. 6d.

"Uniform System of Accounts for Hospitals and Public Institutions." New Edition. 4s. net.

"Hospital Expenditure: The Commissariat." 2s. 6d.

"A Legal Handbook for the Use of Hospital Authorities." 2s. 6d.

"The Cottage Hospital Case Book or Register of Patients." 10s. and 12s. 6d.

All these are published by the Scientific Press, Ltd., and may be obtained through any bookseller, or direct from the publishers, 28 and 29 Southampton Street, Strand, London, W.C.

### NURSING MANUALS.

**THE NURSING PROFESSION: How and Where to Train.**
By Sir Henry Burdett, K.C.B.
2/- net, 2/4 post free.

**THE NURSES' PRONOUNCING DICTIONARY.**
By Honnor Morten.
New Edition.
Edited by M. I. Burdett.
2/- post free.

**PRACTICAL GUIDE TO SURGICAL BANDAGING AND DRESSINGS.**
By W. Johnson Smith, F.R.C.S.
2/- post free.

**ELEMENTARY ANATOMY AND SURGERY FOR NURSES.**
By Wm. McAdam Eccles.
2/6 post free.

**ELEMENTARY PHYSIOLOGY FOR NURSES.**
By C. F. Marshall, M.D.
2/- post free.

**A HANDBOOK FOR NURSES.**
By J. K. Watson, M.D.
5/- net, 5/4 post free.

**NURSING: ITS THEORY AND PRACTICE.**
By Percy Lewis, M.D.
3/6 post free.

THE
**Scientific Press, Ltd.**
**Publish and Stock all descriptions of Nursing Text-Books, Case-Books, Charts, &c.**
*Send a Postcard for New Catalogue.*
**All Enquiries receive Careful and Prompt Attention.**

**NURSING: ITS PRINCIPLES AND PRACTICE.**
By Isabel Adams Hampton.
7/6 net, 7/10 post free.

**SURGICAL WARD WORK AND NURSING.**
By Alex. Miles, M.D.
Contains particulars and Illustrations of the instruments used in various operations.
3/6 net, 3/10 post free.

**A COMPLETE HANDBOOK OF MIDWIFERY.**
By J. K. Watson, M.D.
6/- net, 6/4 post free.

**HOW TO BECOME A CERTIFIED MIDWIFE.**
By Dr. E. Appel.
With full information as to the requirements of the New Act.
1/- net, 1/1 post free.

**MENTAL NURSING.**
By Dr. Wm. Harding.
1/- post free.

**FEVER NURSING.**
By Dr. Wm. Harding.
A valuable guide to Nursing in Fever Cases.
1/- post free.

**ART OF MASSAGE.**
By Mrs. Creighton Hall.
Second Edition. 6/- post free.

**EXAMINATION OF THE URINE.**
By J. K. Watson, M.D.
A valuable little handbook designed for nurses' use.
1/- net, 1/1 post free.

